# Effects of marital status on overall and cancer-specific survival in laryngeal cancer patients: a population-based study

**DOI:** 10.1038/s41598-020-80698-z

**Published:** 2021-01-12

**Authors:** Zhao Ding, Deshun Yu, Hefeng Li, Yueming Ding

**Affiliations:** 1 Clinical Medical College, Dali University, Dali, 671000 Yunnan China; 2 Department of Otolaryngology, The First Affiliated Hospital of Dali University, Dali, 671000 Yunnan China

**Keywords:** Cancer, Psychology

## Abstract

Marital status has long been recognized as an important prognostic factor for many cancers, however its’ prognostic effect for patients with laryngeal cancer has not been fully examined. We retrospectively analyzed 8834 laryngeal cancer patients in the Surveillance Epidemiology and End Results database from 2004 to 2010. Patients were divided into four groups: married, widowed, single, and divorced/separated. The difference in overall survival (OS) and cancer-specific survival (CSS) of the various marital subgroups were calculated using the Kaplan–Meier curve. Multivariate Cox regression analysis screened for independent prognostic factors. Propensity score matching (PSM) was also conducted to minimize selection bias. We included 8834 eligible patients (4817 married, 894 widowed, 1732 single and 1391 divorced/separated) with laryngeal cancer. The 5-year OS and CSS of married, widowed, single, and separated/divorced patients were examined. Univariate and multivariate analyses found marital status to be an independent predictor of survival. Subgroup survival analysis showed that the OS and CSS rates in widowed patients were always the lowest in the various American Joint Committee on Cancer stages, irrespective of sex. Widowed patients demonstrated worse OS and CSS in the 1:1 matched group analysis. Among patients with laryngeal cancer, widowed patients represented the highest-risk group, with the lowest OS and CSS.

## Introduction

Laryngeal cancer has the highest incidence in head and neck cancer, accounting for approximately 1–5% of global cancer incidence^1^. The majority of the patients with laryngeal cancer are middle-aged and elderly men over 40 years of age, with a slightly younger trend^2^. Surgery remains the primary treatment for laryngeal cancer, although the status of radiotherapy, chemotherapy, and a new targeted therapy is gradually rising^3^. For example, a recent study showed that definite radiotherapy is the preferred treatment regardless of the characteristics of the tumor^4^. However, the survival rate was unsatisfactory. Therefore, it is crucial to explore factors affecting the prognosis of patients with laryngeal cancer.

In the past, most cancer research focused on biology, and social or psychological factors were easily ignored. Fortunately, studies on the influence of marital status on the prognosis of cancer have gradually attracted widespread attention. Many studies have confirmed that marital status may affect the prognosis of various types of cancer, including endometrial cancer^5^, ovarian cancer^6^, glioblastoma multiforme^7^, chondrosarcoma^8^ and male breast cancer^9^. Nevertheless, currently there has been limited research on the relationship between laryngeal cancer prognosis and marital status. This study explored the effect of marital status on the survival rate of patients with laryngeal cancer by analyzing data from the Surveillance Epidemiology and End Results (SEER) database.

## Results

### Demographic characteristics

**Figure 1:**
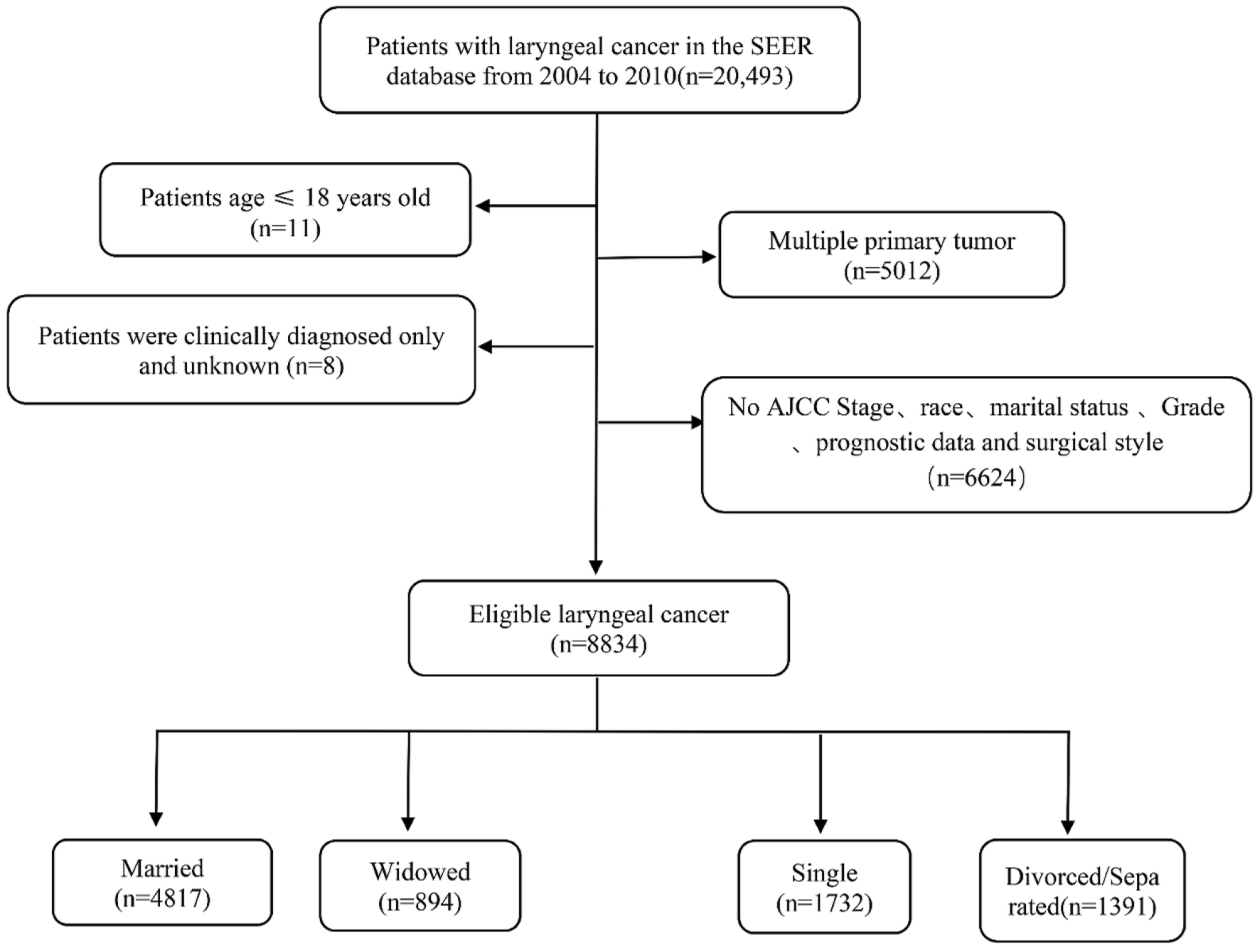
Flow chart for screening eligible patients.

The cohort included a total of 8,834 eligible cases of laryngeal cancer diagnosed between 2004 and 2010. The exact screening process is shown in Fig. 1. The baseline characteristics of eligible patients and the relationship between marital status and variables are shown in Table 1. Among these, 54.5% (n = 4817) patients were married, 10.1% (n = 894) were widowed, 19.6% (n = 1732) were single, and 15.8% (n = 1391) were divorce/separated. The differences in demographic and pathological characteristics between the married group and the other three groups were statistically significant in terms of sex, age, race, grade, surgery, American Joint Committee on Cancer (AJCC) stage, radiotherapy, and chemotherapy (*P* < 0.001). However, there were no significant differences in histological types between the married group and the other three groups (*P* = 0.950).

**Table 1:** Baseline clinicopathological characteristics of patients.

Characteristics	Total	Married	Widowed	Single	Divorced/separated	P value
	N = 8834	N = 4817	N = 894	N = 1732	N = 1391	
**Sex**						< 0.001
Male(%)	7166	4151 (86.2)	494(55.3)	1417(81.8)	1104 (79.4)	
Female(%)	1668	666 (13.8)	400 (44.7)	315(18.2)	287 (20.6)	
**Age**						< 0.001
< 65(%)	5005	2580 (53.6)	201(22.5)	1298(74.9)	926 (66.6)	
≥ 65(%)	3829	2237 (46.4)	693(77.5)	434(25.1)	465 (33.4)	
**Race**						< 0.001
White(%)	7132	4103 (85.2)	717(80.2)	1176(67.9)	1136(81.7)	
Black(%)	1378	474 (9.8)	153(17.1)	521(30.1)	230(16.5)	
Other^1^(%)	324	240 (5.0)	24(2.7)	35(2.1)	25(1.8)	
**Grade**						< 0.001
Grade I(%)	1490	911 (18.9)	120(13.4)	247(14.3)	212(15.2)	
Grade II(%)	5155	2827 (58.7)	524(58.6)	1025(59.2)	779(56.0)	
Grade III/ Grade IV(%)	2189	1079 (22.4)	250(28.0)	460(26.5)	400(28.8)	
**Histological type**						0.95
Squamous cell carcinoma(%)	8563	4669 (96.9)	869(97.2)	1679(96.9)	1346(96.8)	
Othe^r2^(%)	271	148 (3.1)	25(2.8)	53(3.1)	45(3.2)	
**Surgery**						< 0.001
No (%)	5345	2816 (58.5)	599(67.0)	1055(60.9)	875(62.9)	
Yes (%)	3489	2001 (41.5)	295(33.0)	677(39.1)	516(37.1)	
**AJCC stage**						< 0.001
I (%)	2885	1906 (39.6)	249(27.9)	394(22.7)	336(24.2)	
II (%)	1497	861(17.9)	178(19.9)	232(13.4)	226(16.2)	
III (%)	1657	832 (17.3)	189(21.1)	343(19.8)	293(21.0)	
IV (%)	2795	1218 (25.2)	278(31.1)	763(44.1)	536(38.5)	
**Radiotherapy**						< 0.001
Yes (%)	2566	1474 (30.6)	213(23.8)	494(28.5)	385(27.7)	
No/unknown (%)	6268	3343(69.4)	681(76.2)	1238(71.5)	1006(72.3)	
**Chemotherapy**						< 0.001
Yes (%)	3196	1562 (32.4)	279(31.2)	747(43.1)	608(43.7)	
No/unknown (%)	5638	3255(67.6)	615(68.8)	985(56.9)	783(56.3)	

Other^1^ (Asian or Pacific Islander, American Indian/Alaska Native).

Other^2^ (Neoplasm, malignant; Carcinoma, NOS; Carcinoma, undifferentiated, NOS; Large cell carcinoma, NOS; Large cell neuroendocrine carcinoma; Spindle cell carcinoma, NOS; Pseudosarcomatous carcinoma; Carcinoma, anaplastic, NOS; Non-small cell carcinoma; Small cell carcinoma, NOS; Papillary squamous cell carcinoma; Verrucous carcinoma, NOS; Combined small cell carcinoma; Papillary carcinoma, NOS; Basaloid squamous cell carcinoma; Lymphoepithelial carcinoma; Basaloid carcinoma; Adenocarcinoma, NOS; Scirrhous adenocarcinoma; Adenoid cystic carcinoma; Neuroendocrine carcinoma, NOS; Mucoepidermoid carcinoma; Papillary carcinoma, follicular variant; Adenosquamous carcinoma; Adenocarcinoma with cartilaginous and osseous metaplasia; Adenocarcinoma with neuroendocrine differentiation; Carcinoma in pleomorphic adenoma).

The widowed group had the highest proportion of women, the highest number of elderly patients (≥ 65 years), the highest number of AJCC II/III tumors, and the highest proportion of untreated patients (surgery, radiotherapy, and chemotherapy), which was significantly different from the other marital status groups (*P* < 0.001). Compared with patients who were widowed (33.0%), single (39.1%), or divorced/separated (37.1%), married (41.5%) patients were more likely to have surgery (*P* < 0.001).

### Influence of marriage status on overall survival (OS) of laryngeal cancer in SEER database

**Figure 2:**
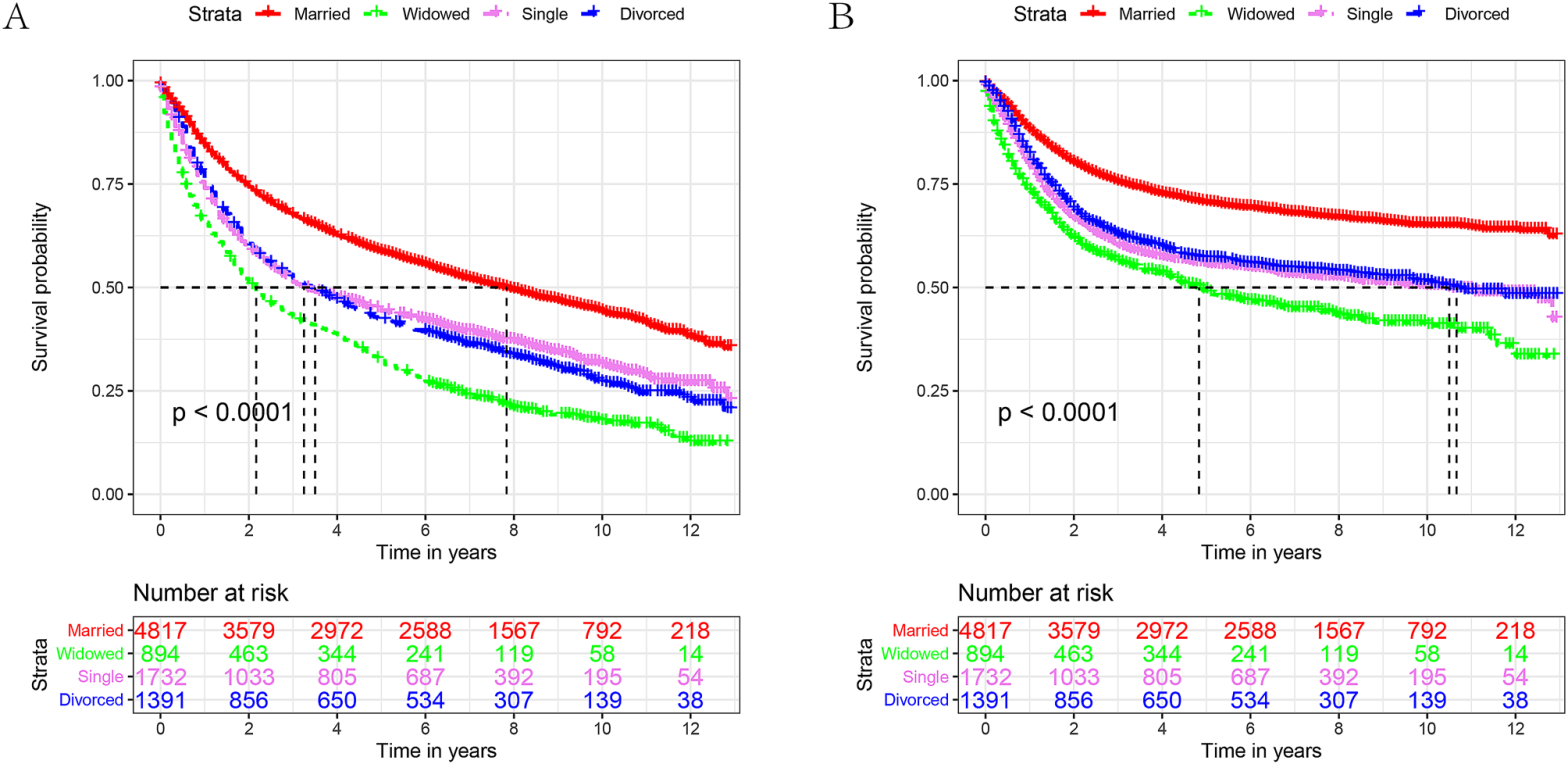
Kaplan–Meier survival curves for OS (A) and CSS (B) in different marital statuses. (OS: overall survival, CSS: cause-specific survival).

Kaplan–Meier survival analysis showed significant differences in OS results between the various marital status (*P* < 0.001, Fig. 2A). The married group had the highest 5-year OS (58.6%) compared to the other groups. The 5-year OS of patients with laryngeal cancer in the widowed group was the lowest (32.3%). After the univariate log-rank test, all differences were significant except for sex (*P* < 0.001), Table 2. Multivariate Cox regression indicated that age, race, grade, histological type, surgery, AJCC stage, chemotherapy, and marital status were independent prognostic factors affecting survival. However, radiotherapy was not an independent prognostic factor affecting survival (Table 2). Cox regression analysis showed that, compared with married patients, the risk of widowed, single (HR: 1.34, 95% CI: 1.24–1.44), divorced/separated (HR: 1.36, 95% CI: 1.27–1.47) was higher, and widowed patients (HR: 1.62, 95% CI: 1.49–1.77) had the highest risk of death.

**Table 2:** Univariate and multivariate survival analysis of OS in laryngeal cancer patients before PSM.

Variable	5-year OS	Univariate analysis		Multivariate analysis	
		Log-rank χ2 test	P	HR (95% CI)	P
**Sex**		2.552	0.11		
Male	50.90%				
Female	48.90%				
**Age**		295.001	< 0.001		P < 0.001
< 65	56.30%			Reference	
≥ 65	43.10%			1.87(1.76–1.98)	
**Race**		64.141	< 0.001		P < 0.001
White	51.70%			Reference	
Black	42.30%			1.11(1.03–1.19)	
Other^1^	60.00%			0.80(0.68–0.94)	
**Grade**		330.634	< 0.001		P < 0.001
Grade I	66.40%			Reference	
Grade II	51.70%			1.22(1.12–1.33)	
Grade III/ Grade IV	37.00%			1.43(1.30–1.57)	
**Histological type**		21.933	< 0.001		0.007
Squamous cell carcinoma	50.90%			Reference	
Other^2^	40.10%			1.21(1.05–1.40)	
**Surgery**		109.405	< 0.001		P < 0.001
No	46.30%			Reference	
Yes	57.00%			0.70(0.64–0.76)	
**AJCC stage**		1173.743	< 0.001		P < 0.001
I	71.10%			Reference	
II	56.70%			1.47(1.34–1.61)	
III	43.60%			2.20(2.01–2.41)	
IV	30.10%			3.38(3,11–3.68)	
**Radiotherapy**		20.714	< 0.001		0.813
Yes	53.80%			Reference	
No/unknown	49.20%			1.01(0.92–1.10)	
**Chemotherapy**		259.862	< 0.001		P < 0.001
Yes	39.70%			Reference	
No/unknown	56.70%			1.14(1.07–1.23)	
**Marital status**		432.26	< 0.001		P < 0.001
Married	58.60%			Reference	
Widowed	32.30%			1.62(1.49–1.77)	
Single	44.30%			1.34(1.24–1.44)	
Divorced/Separated	42.20%			1.36(1.27–1.47)	

Other^1^ (Asian or Pacific Islander, American Indian/Alaska Native).

Other^2^ (Neoplasm, malignant; Carcinoma, NOS; Carcinoma, undifferentiated, NOS; Large cell carcinoma, NOS; Large cell neuroendocrine carcinoma; Spindle cell carcinoma, NOS; Pseudosarcomatous carcinoma; Carcinoma, anaplastic, NOS; Non-small cell carcinoma; Small cell carcinoma, NOS; Papillary squamous cell carcinoma; Verrucous carcinoma, NOS; Combined small cell carcinoma; Papillary carcinoma, NOS; Basaloid squamous cell carcinoma; Lymphoepithelial carcinoma; Basaloid carcinoma; Adenocarcinoma, NOS; Scirrhous adenocarcinoma; Adenoid cystic carcinoma; Neuroendocrine carcinoma, NOS; Mucoepidermoid carcinoma; Papillary carcinoma, follicular variant; Adenosquamous carcinoma; Adenocarcinoma with cartilaginous and osseous metaplasia; Adenocarcinoma with neuroendocrine differentiation; Carcinoma in pleomorphic adenoma).

OS, overall survival; PSM, propensity score matching; HR, hazard ratio; CI, confidence interval.

### Effect of marital status on cancer-specific survival (CSS) survival

To explore the correlation between marital status and CSS, we performed Kaplan–Meier survival analysis on patients in the dataset. Figure 2B shows a significant difference in CSS among laryngeal cancer patients with various marital statuses. Male sex (*P* = 0.004), age < 65 years (*P* < 0.001), race (*P* < 0.001), grade I (*P* < 0.001), squamous cell carcinoma (*P* < 0.001), yes for surgery (*P* < 0.001), AJCC Stage I (*P* < 0.001), yes for radiotherapy (*P* = 0.001), no or unknown for chemotherapy (*P* = 0.001), and married state (*P* < 0.001) were associated with a higher 5-year CSS. (Table 3). To prevent possible interference between the variables, we used multivariate Cox regression analysis. As with the OS results, radiotherapy was not an independent predictor of CSS in patients with laryngeal cancer. In terms of marital status, married state remains a protective factor for the prognosis of laryngeal cancer.

**Table 3:** Univariate and multivariate survival analysis of CSS in laryngeal cancer patients before PSM.

Variable	5-year CSS	Univariate analysis		Multivariate analysis	
		Log-rank χ2 test	P	HR (95% CI)	P
**Sex**		8.267	0.004		0.009
Male	64.00%			Reference	
Female	61.10%			0.88(0.81–0.97)	
**Age**		41.735	< 0.001		P < 0.001
< 65	65.80%			Reference	
≥ 65	60.30%			1.55(1.44–1.67)	
**Race**		57.35	< 0.001		0.037
White	64.90%			Reference	
Black	54.40%			1.10(1.01–1.21)	
Other^1^	68.70%			0.88(0.73–1.08)	
**Grade**		393.314	< 0.001		P < 0.001
Grade I	79.60%			Reference	
Grade II	65.10%			1.38(1.23–1.56)	
Grade III/ Grade IV	48.40%			1.68(1.48–1.92)	
**Histological type**		31.108	< 0.001		0.002
Squamous cell carcinoma	63.90%			Reference	
Other^2^	49.70%			0.76(0.64–0.90)	
**Surgery**		107.929	< 0.001		P < 0.001
No	59.40%			Reference	
Yes	69.60%			0.61(0.55–0.68)	
**AJCC stage**		1551.781	< 0.001		P < 0.001
I	85.70%			Reference	
II	72.50%			1.84(1.61–2.10)	
III	56.80%			3.32(2.93–3.78)	
IV	38.70%			5.94(5.27–6.70)	
**Radiotherapy**		10.406	0.001		0.729
Yes	65.50%			Reference	
No/unknown	62.60%			0.98(0.87–1.09)	
**Chemotherapy**		409.833	< 0.001		0.002
Yes	49.60%			Reference	
No/unknown	71.40%			1.14(1.05–1.24)	
**Marital status**		288.866	< 0.001		P < 0.001
Married	70.50%			Reference	
Widowed	49.30%			1.70(1.52–1.91)	
Single	55.50%			1.33(1.21–1.46)	
Divorced/separated	56.90%			1.27(1.15–1.40)	

Other^1^ (Asian or Pacific Islander, American Indian/Alaska Native).

Other^2^ (Neoplasm, malignant; Carcinoma, NOS; Carcinoma, undifferentiated, NOS; Large cell carcinoma, NOS; Large cell neuroendocrine carcinoma; Spindle cell carcinoma, NOS; Pseudosarcomatous carcinoma; Carcinoma, anaplastic, NOS; Non-small cell carcinoma; Small cell carcinoma, NOS; Papillary squamous cell carcinoma; Verrucous carcinoma, NOS; Combined small cell carcinoma; Papillary carcinoma, NOS; Basaloid squamous cell carcinoma; Lymphoepithelial carcinoma; Basaloid carcinoma; Adenocarcinoma, NOS; Scirrhous adenocarcinoma; Adenoid cystic carcinoma; Neuroendocrine carcinoma, NOS; Mucoepidermoid carcinoma; Papillary carcinoma, follicular variant; Adenosquamous carcinoma; Adenocarcinoma with cartilaginous and osseous metaplasia; Adenocarcinoma with neuroendocrine differentiation; Carcinoma in pleomorphic adenoma).

CSS, cancer-specific survival; PSM, propensity score matching; HR, hazard ratio; CI, confidence interval.

### Subgroup analysis by AJCC stage

**Figure 3:**
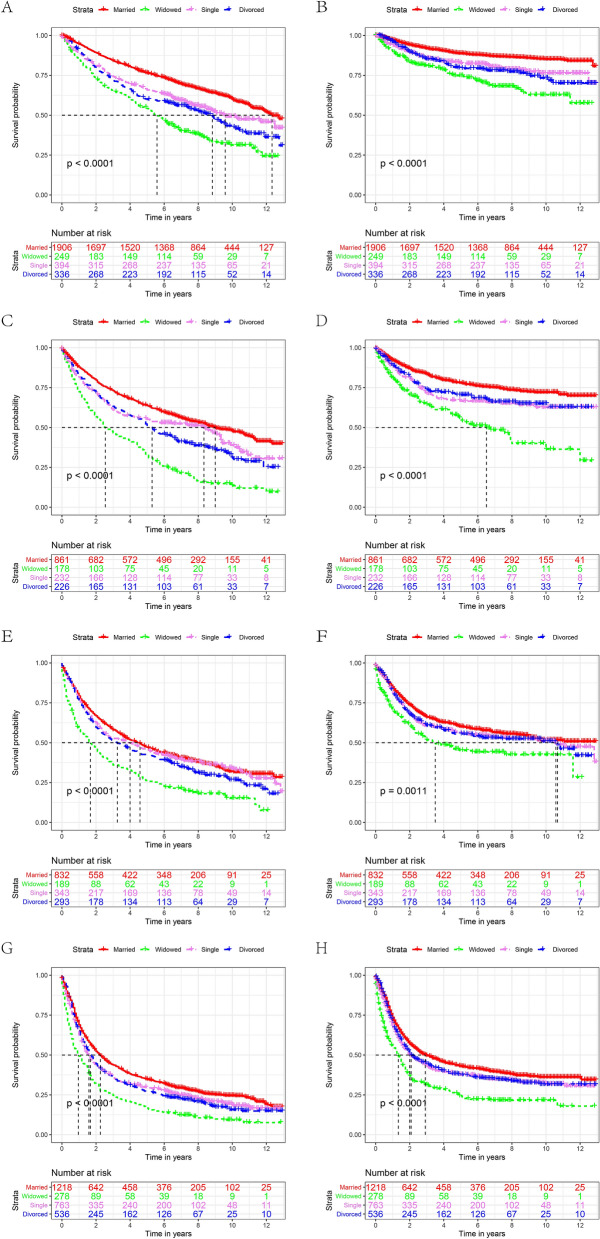
The OS and CSS curves of laryngeal cancer patients with different AJCC stages. A and B, OS and CSS for StageI. C and D, OS and CSS for StageII. E and F, OS and CSS for StageIII. G and H, OS and CSS for StageIV. (OS: overall survival, CSS: cause-specific survival).

We further evaluated the impact of marital status on OS and CSS of each AJCC stage. Interestingly, we obtained similar results in the various AJCC stage subgroups (Tables 4, 5, Fig. 3). First, marital status was an independent factor affecting the OS and CSS of each AJCC stage in univariate and multivariate analyses (*P* < 0.001). Second, the 5-year OS and CSS of patients in the widowed group were consistently lower than those of the other marital groups (Fig. 3). In terms of OS, among all AJCC stages, the survival rate of widowed patients in the AJCC stage II group was significantly lower than that of married patients (32.6% vs. 63.7%, *P* < 0.001; HR 2.06, 95% CI 1.70. 2.50, *P* < 0.001, Table 4). In the analysis of CSS, the most significant difference was between widowed patients and married patients in the AJCC stage III subgroup (32.3% vs. 58.6%, *P* < 0.001; HR 1.28, 95% CI 1.00.1.64, *P* = 0.042, Table 5). This phenomenon also occurred in the AJCC stage III subgroup. The risk of death was higher in the divorced/separated group than in the married group (HR 1.19; 95% CI, 0.97. 1.46), although the difference was not significant (*P* = 0.092, Table 5).

**Table 4:** Univariate and multivariate analysis of the effect of marital status on laryngeal cancer OS based on different cancer stages before PSM.

Variable	5-year OS	Univariate analysis		Multivariate analysis	
		Log-rank χ2 test	P	HR (95% CI)	P
**AJCC stage**					
**Stage I**					
**Marital Status**		119.59	< 0.001		
Married	76.40%			Reference	
Widowed	54.20%			1.67(1.40–2.00)	P < 0.001
Single	65.80%			1.61(1.37–1.91)	P < 0.001
Divorced/separated	59.80%			1.81(1.53–2.13)	P < 0.001
**Stage II**					
**Marital status**		103.231	< 0.001		
Married	63.70%			Reference	
Widowed	32.60%			2.06(1.70–2.50)	P < 0.001
Single	55.10%			1.47(1.20–1.81)	P < 0.001
Divorced/separated	51.30%			1.52(1.26–1.84)	P < 0.001
**Stage III**					
**Marital status**		48.854	< 0.001		
Married	47.20%			Reference	
Widowed	26.40%			1.45(1.21–1.74)	P < 0.001
Single	45.60%			1.19(1.01–1.40)	0.032
**Divorced/separated**	42.30%			1.25(1.06–1.47)	0.006
**Stage IV**					
**Marital status**		68.915	< 0.001		
Married	34.70%			Reference	
Widowed	16.70%			1.44(1.24–1.66)	P < 0.001
Single	29.60%			1.17(1.05–1.30)	0.004
Divorced/separated	27.20%			1.21(1.07–1.36)	0.001

OS, overall survival; PSM, propensity score matching; HR, hazard ratio; CI, confidence interval.

**Table 5:** Univariate and multivariate analysis of the effect of marital status on laryngeal cancer CSS based on different cancer stages before PSM.

Variable	5-year CSS	Univariate analysis		Multivariate analysis	
		Log-rank χ2 test	P	HR (95% CI)	P
**AJCC stage**					
**Stage I**					
**Marital status**		72.874	< 0.001		
Married	88.70%			Reference	
Widowed	74.10%			2.24(1.68–2.99)	P < 0.001
Single	82.60%			1.67(1.28–2.18)	P < 0.001
Divorced/separated	79.20%			1.92(1.47–2.51)	P < 0.001
**Stage II**					
**Marital status**		53.982	< 0.001		
Married	77.70%			Reference	
Widowed	56.00%			2.24(1.70–2.94)	P < 0.001
Single	66.40%			1.65(1.25–2.71)	P < 0.001
Divorced/separated	70.40%			1.38(1.04–1.82)	0.022
**Stage III**					
**Marital status**		16.011	0.001		
Married	58.60%			Reference	
Widowed	32.30%			1.28(1.00–1.64)	0.042
Single	44.30%			1.28(1.05–1.56)	0.014
Divorced/separated	42.20%			1.19(0.97–1.46)	0.092
**Stage IV**					
**Marital status**		53.499	< 0.001		
Married	43.00%			Reference	
Widowed	24.30%			1.58(1.33–1.86)	P < 0.001
Single	37.70%			1.17(1.03–1.32)	0.012
Divorced/separated	37.60%			1.17(1.02–1.33)	0.022

CSS, cancer-specific survival; PSM, propensity score matching; HR, hazard ratio; CI, confidence interval.

**Table 6:** Univariate and multivariate analysis of the effect of marital status on laryngeal cancer OS based on different sex before PSM.

Variable	5-year OS	Univariate analysis		Multivariate analysis	
		Log-rank χ2 test	P	HR (95% CI)	P
**Sex**					
**Male **					
Marital status		341.657	< 0.001		
Married	58.50%			Reference	
Widowed	31.60%			1.68(1.51-1.87)	P < 0.001
Single	43.20%			1.39(1.28-1.50)	P < 0.001
Divorced/Separated	41.00%			1.43(1.32-1.56)	P < 0.001
**Female**					
Marital Status		101.730	< 0.001		
Married	59.00%			Reference	
Widowed	33.10%			1.61(1.37-1.89)	P < 0.001
Single	49.50%			1.15(0.95-1.38)	0.146
Divorced/separated	46.80%			1.19(0.99-1.42)	0.062

OS, overall survival; PSM, propensity score matching; HR, hazard ratio; CI, confidence interval.

**Table 7:** Univariate and multivariate analysis of the effect of marital status on laryngeal cancer CSS based on different sex before PSM.

Variable	5-year CSS	Univariate analysis		Multivariate analysis	
		Log-rank χ2 test	P	HR (95% CI)	P
**Sex**					
**Male**					
Marital status		230.951	< 0.001		
Married	70.80%			Reference	
Widowed	51.30%			1.63(1.41–1.88)	P < 0.001
Single	54.40%			1.36(1.23–1.51)	P < 0.001
Divorced/separated	55.40%			1.36(1.25–1.52)	P < 0.001
**Female**					
Marital status		64.624	< 0.001		
Married	68.60%			Reference	
Widowed	45.60%			1.57(1.29–1.91)	P < 0.001
Single	60.20%			1.13(0.90–1.42)	0.293
Divorced/separated	62.30%			0.94(0.75–1.19)	0.620

CSS, cancer-specific survival; PSM, propensity score matching; HR, hazard ratio; CI, confidence interval.

### Subgroup analysis by sex

**Figure 4:**
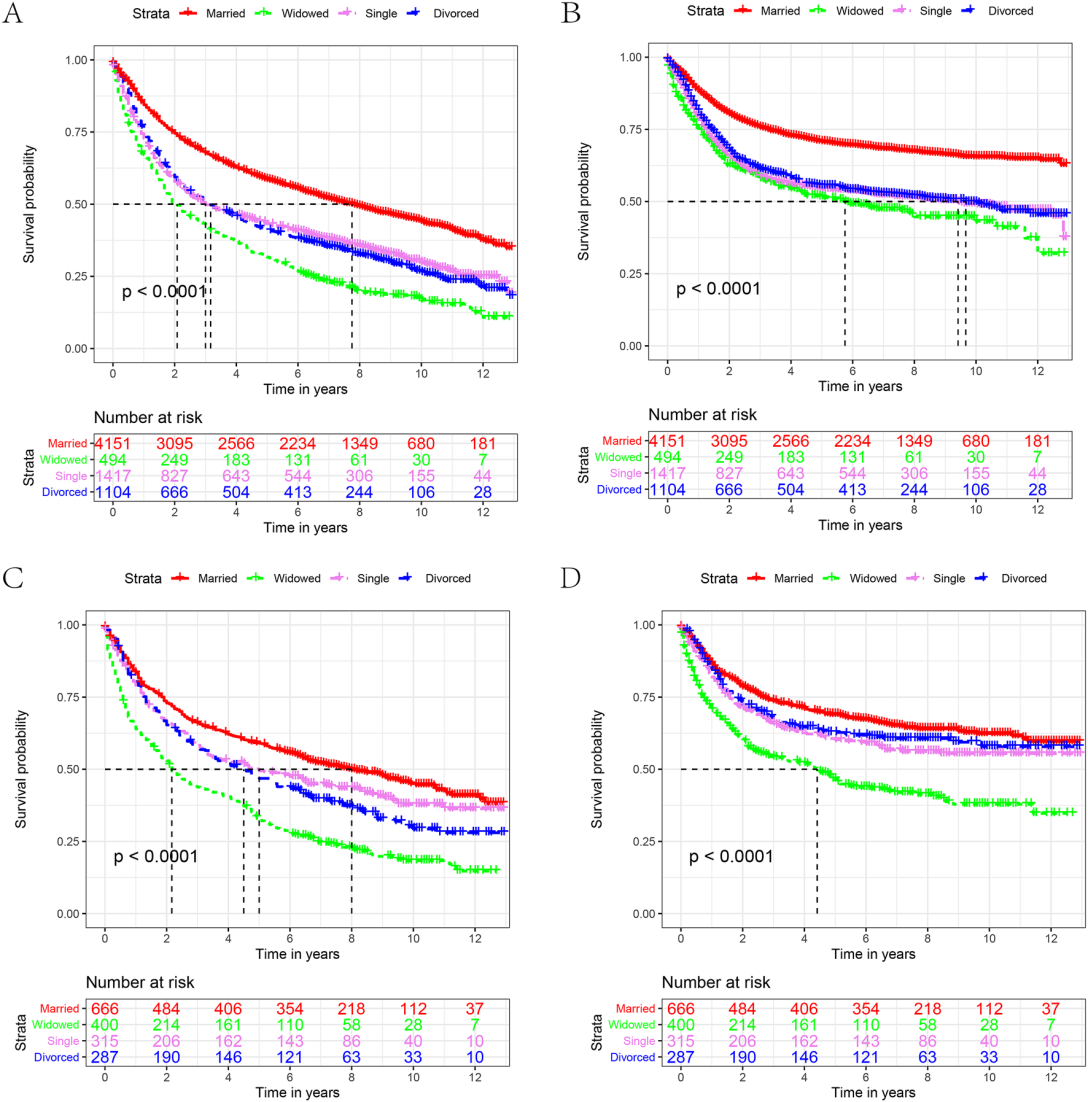
The OS and CSS curves of laryngeal cancer patients with different sex.** A** and** B**, OS and CSS for male.** C** and** D**, OS and CSS for female (OS: overall survival, CSS: cause-specific survival).

Subsequently, we analyzed the influence of marital status on OS and CSS rates for each sex. Figures 4 shows the Kaplan–Meier curve of OS and CSS rates among the sexes. Regardless of the sex, the OS and CSS of the widowed group were lower than those of the other groups. Compared with married patients in the male group, the 5-year OS and CSS of widowed patients were reduced by 26.9% and 19.5%, respectively (58.50% vs. 31.60%, *P* < 0.001, 70.80% vs. 51.30%, *P* < 0.001, Tables 6, 7). Compared with married patients in the subgroup, the reduction was 25.9% and 23.0%, respectively (59.00% vs. 33.10%, *P* < 0.001; 68.60% vs. 45.60%, *P* < 0.001, Tables 6 and 7). In line with the previous results, widowed patients were at the highest risk for death when comparing the OS and CSS among all the groups. (Tables 6 and 7).

### Survival analysis in 1:1 matched group

**Figure 5:**
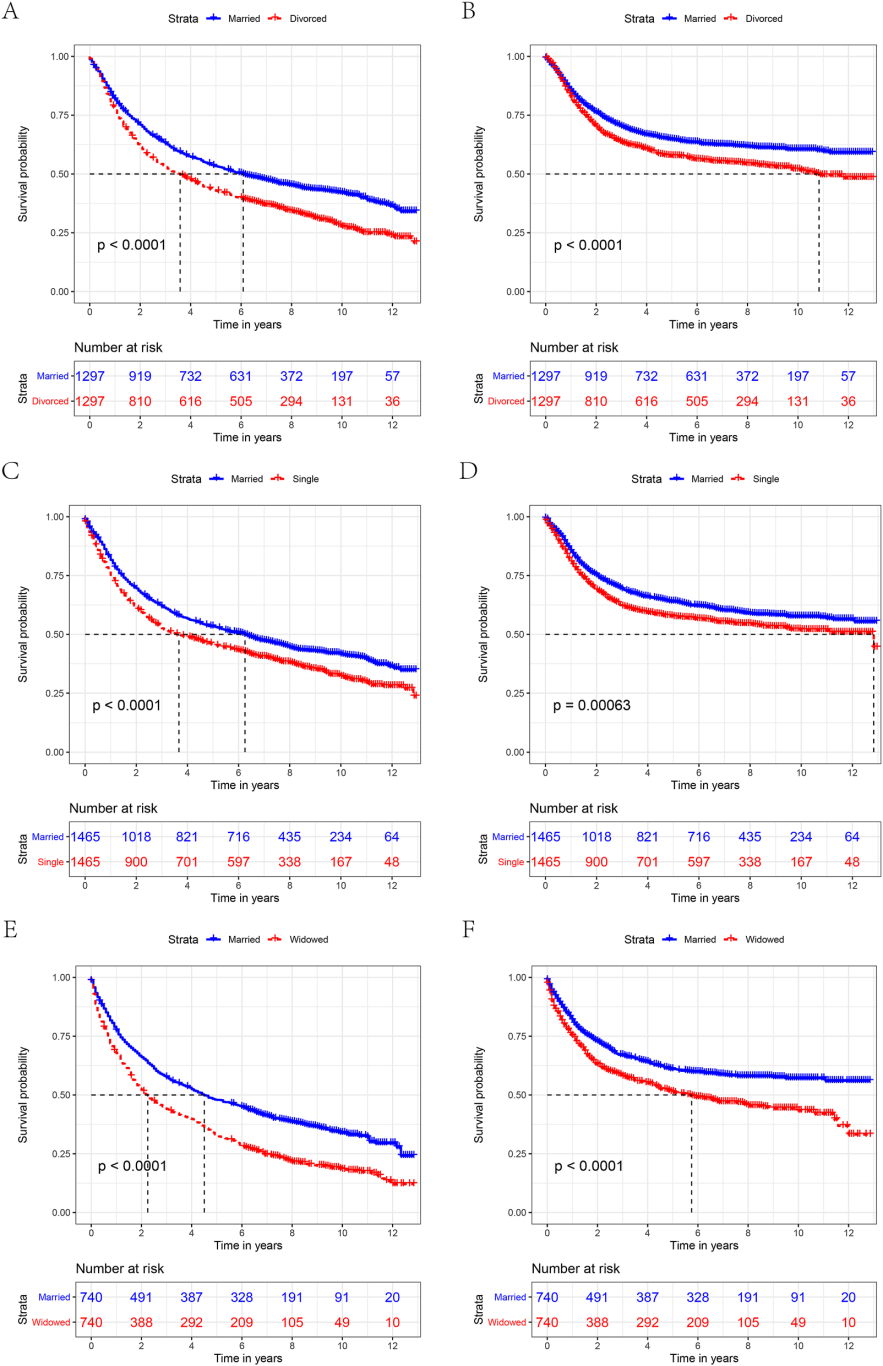
The OS (A, C, E) and CSS (B, D, F) of patients with laryngeal cancer according to marital status after PSM. (OS: overall survival, CSS: cause-specific survival).

To minimize the effect of possible confounding factors on the baseline features of the marriage subgroups and to verify the reliability of our results, we implemented a 1:1 matching cohort utilizing propensity score-matching (PSM) methods. We obtained three 1:1 matched cohorts, including a single and married cohort, a widowed and married cohort, and a divorced/separated and married cohort. The demographic and clinicopathological features of the matched cohort are presented in Table 8. As expected, the clinicopathological parameters were well balanced between the groups after PSM. Widowed patients showed worse OS and CSS in the divorce/separated-married cohort (Fig. 5A,B), the single-married cohort (Fig. 5C,D), and the widowed-married cohort (Fig. 5E,F).

**Table 8:** Patient baseline characteristics after PSM.

Characteristics	Divorced/separated	Married	P value	Single	Married	P value	Widowed	Married	P value
	N = 1297	N = 1297		N = 1465	N = 1465		N = 740	N = 740	
**Sex**			0.723			0.797			0.957
Male (%)	1055(81.3)	1062(81.9)		1239(84.6)	1244(84.9)		477(64.5)	478(64.6)	
Female (%)	242(18.2)	235(18.1)		226(15.4)	221(15.1)		263(35.5)	262(35.4)	
**Age**			1.000			0.645			0.952
< 65(%)	869(67.0)	869(67.0)		1071(73.1)	1081(73.9)		185(25.0)	184(24.9)	
≥ 65(%)	428(33.0)	428(33.0)		394(26.9)	383(26.1)		555(75.0)	556(75.1)	
**Race**			0.969			0.387			0.976
White (%)	1090(84.0)	1094(84.3)		1118(76.3)	1124(76.7)		620(83.8)	622(84.1)	
Black (%)	191(14.7)	188(14.5)		325(22.2)	310(21.2)		107(14.5)	106(14.3)	
Other^1^(%)	16(1.2)	15(1.2)		22(1.5)	31(2.1)		13(1.8)	12(1.6)	
**Grade**			0.998			0.787			0.373
Grade I (%)	195(15.0)	195(15.0)		205(14.0)	215(14.7)		97(13.1)	114(15.4)	
Grade II (%)	756(58.3)	756(58.3)		896(61.2)	899(61.4)		448(60.5)	446(60.3)	
Grade III/grade IV (%)	346(26.7)	347(26.8)		364(24.8)	351(24.0)		195(26.4)	180(24.3)	
**Histological type**			0.892			0.892			0.510
Squamous cell carcinoma (%)	1270(97.9)	1269(97.8)		1437(98.1)	1438(98.2)		731(98.8)	728(98.4)	
Other^2^(%)	27(2.1)	28(2.2)		28(1.9)	27(1.8)		9(1.2)	12(1.6)	
**Surgery**			0.903			0.879			0.060
No (%)	815(62.8)	812(62.6)		897(61.2)	901(61.5)		505(68.2)	538(72.7)	
Yes (%)	482(37.2)	485(37.4)		568(38.8)	564(38.5)		235(31.8)	202(27.3)	
**AJCC stage**			0.998			0.846			0.861
I (%)	331(25.5)	328(25.3)		368(25.1)	388(26.5)		224(30.3)	231(31.2)	
II (%)	207(16.0)	206(15.9)		207(14.1)	202(13.8)		150(20.3)	150(20.3)	
III (%)	270(20.8)	270(20.8)		292(19.9)	281(19.2)		160(21.6)	167(22.6)	
IV (%)	489(37.7)	493(38.0)		598(40.8)	594(40.5)		206(27.8)	192(25.9)	
**Radiotherapy**			0.929			0.621			0.853
Yes (%)	348(26.8)	350(27.0)		413(28.2)	401(27.4)		172(23.2)	169(22.8)	
No/unknown (%)	949(73.2)	947(73.0)		1057(71.8)	1064(72.6)		568(76.8)	571(77.2)	
**Chemotherapy**			0.968			0.681			1.000
Yes (%)	569(43.9)	570(43.9)		622(42.5)	611(41.7)		235(31.8)	235(31.8)	
No/unknown (%)	728(56.1)	727(56.1)		843(57.5)	854(58.3)		505(68.2)	505(68.2)	

PSM, propensity score matching.

## Discussion

For the first time, a population analysis based on the SEER database was performed to assess the prognostic impact of marital status on the survival rate of patients with laryngeal cancer. This study found that marital status is an independent factor affecting the prognosis of laryngeal cancer. More specifically, married patients have the lowest risk of death, while widowed patients have the highest risk of death. This is like many previous research results^10–12^. We further confirmed after PSM that widowed patients had better OS and CSS than divorced, single, or married patients.

In 1977, Engel proposed a new model of biological psychological medicine^13^. He believed that biological factors, psychological elements, and social factors influenced disease progression and outcome^14^. Since then, extensive research has been conducted on the relationship between biological psychological factors and disease^15–17^. The role of biopsychosocial factors in cancer patients has also gradually gained attention^18,19^. A study of women's marital status and mortality rates showed that single patients had higher mortality rates than divorced or widowed patients^20^. Another large-scale survey found that married patients with oral and laryngeal cancer are less likely to have metastases^21^. A Swedish study found that divorce and bereavement are risk factors for esophageal and gastric cancer^22^. The relationship between marital status and prognosis may be influenced by tumor stage, proportion of patients receiving treatment, and social support^23–26^. A higher percentage of married laryngeal cancer patients receive timely treatment, including surgery and adjuvant treatment, which may explain the high survival rate. However, it emphasizes the interrelationship between marital status and survival rather than causality. Therefore, it is necessary to explore how marital status affects the potential mechanism of survival to improve the outcome of patients with laryngeal cancer.

This study showed that marital status was associated with survival in patients with laryngeal cancer. We hypothesize the following reasons for the beneficial effect of married state on survival in patients with laryngeal cancer. First, a happy marriage may result in a well-balanced emotional state, and a wholesome family environment may ease work and social pressures. Second, married patients that have stable marriages usually are accompanied by appropriate family finances. A commonly accepted explanation of why married people have lower cancer mortality, was that it was related to better socioeconomic status. This was believed to buffer the effects of stressful events^27,28^. Chronic stress may cause long-term secretion of cortisol^29^, which leads to reverse regulation of leukocytes by down-regulating the cortisol receptor of leukocytes. This downregulation, in turn, reduces the ability of cells to respond to anti-inflammatory signals and leads to the vigorous development of cytokine-mediated inflammatory processes^30^, which has been proven to be a poor prognostic factor for cancer^31,32^. Third, married patients have a wider social range than unmarried patients. They have a broader information base regarding medical equipment, experts, and treatment methods. This can help improve treatment outcomes^33^. Social networks influence patient compliance, and good compliance ultimately affects a patients' health outcomes^34^.

In addition, our results raise another intriguing question as to why widowed patients exhibit worse clinical outcomes. There is evidence to suggest that a widows' health is a problem before they are diagnosed with cancer^35^. Studies have shown that the recent death of a husband results in a significant decrease in the level of natural killer (NK) cells in the widows’ body^36,37^. More importantly, NK cells are known to play an important role in the fight against cancer^38^. Compared with married patients, widowed patients had more psychological stress and less psychological support. This can cause disorders of the immune system and promote cancer progression^39^. Such an alteration affects the release of glucocorticoids and catecholamines, further affecting the tumor microenvironment^40,41^. An enhanced development of tumors results in a shortened survival time for widowed patients.

Although this study is both instructive and relevant to clinical practice, it has some limitations. First, the marital status information provided in the SEER database is incomplete. It provides only marital status at the time of diagnosis, and some patients' marital status may change during follow-up. In the same way, it does not reflect whether marital status changes after diagnosis, which may also affect survival outcomes. In addition, patients aged 65 and above account for approximately 44% of the total. This means that it is possible to change from a married status to a widowed status during the follow-up period. Second, in addition to marital status, various other social factors are included in the bio-psycho-social medical model, such as education, income, and insurance. The SEER database lacks information regarding other societal factors. Our analysis cannot correct these factors, which may affect the survival outcomes. Third, the SEER database does not provide information on whether married patients are happy or not. Even in an equivalent married group of patients, the happiness of marriage may be questionable. For instance, prolonged marital discord can damage the immune system and have a negative effect on health^42^. Fourth, personal history such as smoking, drinking, and human papilloma virus (HPV) infections is not reflected in the SEER database. Some studies have shown that unmarried people may be more likely to engage in the bad habits of smoking and/or drinking^43,44^. Fifth, some patients may live with a partner without being married, despite the fact that the proportion of people in this circumstance may be small. In addition, we classify these patients as single patients, however their survival outcomes are expected to be better than those of other single people, which confounds our results. Sixth, the SEER database does not contain the number of patients with alternate sexual orientation. Finally, the AJCC stage provided in the SEER database is the sixth edition, and now the eighth edition is used in the clinic. The subgroup results of our AJCC stage need to be verified in further clinical practice.

Despite these limitations, our findings are credible for the following reasons. First, our data is from the SEER database, which is composed of data from multiple centers across the country. Second, we chose the two outcomes of OS and CSS, which adds to the accuracy of our results. Last but not least, we also performed a stratified analysis on the AJCC stage and obtained high survival rates for married patients and low survival rates for the widowed patients. Due to a lack of social and psychological support, widowed patients should be advised to obtain psychological therapy during cancer treatment to improve their prognosis.

## Conclusion

Marital status was associated with survival (OS and CSS) in patients with laryngeal cancer. It is clear that married patients had a better survival outcome, while widowed patients had a worse prognosis. If we are able to fully understand the impact of marital status on cancer patients, we will be able to provide individualized treatment and improve survival outcomes.

## Material and methods

### Patients

We utilized the SEER database access online. The reference number 16606-nov2018 was approved for use for accessing the database. From the SEER database (http://seer.cancer.gov), we screened information on 8,834 patients who met the diagnostic criteria for primary laryngeal cancer between 2004 and 2010. The patient inclusion criteria consisted of: (1) age ≥ 18 years age at diagnosis; (2) solitary primary tumor; and (3) histologic type: ICD-O-3 morphology code (8000/3, 8010/3, 8012/3, 8013/3, 8020/3, 8021/3, 8032/3, 8033/3, 8041/3 , 8045/3, 8046/3, 8050/3, 8051/3, 8052/3, , 8070/3, 8071/3, 8072/3, 8073/3, 8074/3, 8075/3, 8076/3, 8078/3, 8082/3, 8083/3, 8123/3, 8140/3, 8141/3, 8200/3, 8246/3, 8340/3, 8430/3, 8560/3, 8571/3, 8574/3, 8941/3). Patients were excluded if: (1) the marital status was unclear; (2) there was a lack of some crucial clinicopathological information, such as AJCC stage, grade, race, and surgical style; (3) they were only diagnosed clinically; and (4) there was an absence of prognostic information.

### Description of covariates

The study variables included the following: sex (male, female), age (< 65 or ≥ 65 years), race (White, Black, or other), Grade (Grade I, Grade II ,Grade III/IV), histological type (squamous cell carcinoma, other), surgery (yes or no), AJCC stage (I, II, III, IV), radiotherapy (yes or no/unknown), chemotherapy (yes or no/unknown), marital status (married, widowed, single, divorced/separated). The laryngeal specific surgical code 10–90 was defined as invasive treatment and code 00 as non-surgical treatment. The primary outcome indicators were laryngeal cancer OS and CSS.

### Statistical analysis

The clinical and pathological characteristics of patients with various marital statuses were compared using the chi-square test (χ^2^). Kaplan–Meier estimates and log-rank tests were used to estimate and compare survival functions between the different variables. Multivariate Cox proportional risk regression was used to estimate the risk ratio (HR) and 95% confidence interval (CI) for OS and CSS in the various marital statuses. To reduce potential baseline confounding factors, we employed propensity score matching (PSM) to re-examine the impact of marital status^45^. One- to- one PSM was conducted using the nearest-neighbor algorithm with a caliper width of 0.2. Data were analyzed using SPSS version 25 (IBM Statistics, New York, NY), and the survival curves were generated by R project version 3.6.1. *P* < 0.05 (2-sided) was considered significant.

## Data Availability

The data used in this study were provided by the SEER database.
